# Neuroplasticity Changes of Rat Brain by Musical Stimuli during Fetal Period

**DOI:** 10.22074/cellj.2015.490

**Published:** 2015-01-13

**Authors:** Siamak Sheikhi, Ehsan Saboory

**Affiliations:** 1 Division of Neuropsychology, Faculty of Medicine, Urmia University of Medical Sciences, Urmia, Iran; 2 Neurophysiology Research Center, Urmia University of Medical Sciences, Urmia, Iran; 3 Department of Physiology, Faculty of Medicine, Urmia University of Medical Sciences, Urmia, Iran

**Keywords:** Fetus, Brain, Neuroplasticity, Music, Gestation

## Abstract

**Objective:**

Fetal development of the central nervous system is an important and sensitive stage which is affected by many external and internal stimuli. This study aimed to investigate effect of musical stimuli on fetal rat brain.

**Materials and Methods:**

In this experimental study, twelve female Wistar rats were selected and evenly assigned to control and musical groups. The females were mated with a male rat of the same genotype. Musical group was exposed to classic music with 60 dB power for 90 minutes twice per day from 2^nd^ to 20^th^ day of gestation. The control rats were handled similar to the musical group, but were not exposed to music. Before parturition, all the dams were anesthetized, and their blood samples were obtained and used for corticosterone (COS) measurement. They were transcardially perfused by electron microscope (EM) fixative agent. The fetal brains were extracted intact and used for slice preparation. Horizontal slices were made for electron microscope preparation, and images were taken and analyzed in terms of cell density and morphological changes.

**Results:**

EM observation indicated significant morphological difference in cellular and intercellular spaces between the two groups. Music-treated fetuses had significantly higher cell density in parietal cortex and music-treated dams had lower COS level.

**Conclusion:**

It was concluded that prenatal music would have a great impact on neuroplasticity of fetal rat brain, at least indirectly. Although the rat fetuses cannot hear until birth, music-induced reduction in COS blood level of dams might be the reason for neuroplasticity of fetal brain.

## Introduction

It has been reported that many factors, including genetic and epigenetic ones, affect development and physical structure of brain (1). Studies have reported that severe stress experienced early in life can extensively impact brain tissue, which indicates reduced volumes and attenuated development of several neural structures (2). During the prenatal period, development of individuals is influenced by environmental factors. Various physical and emotional stresses in mothers lead to low birth weight (LBW) of the offspring, increased risk of premature delivery and higher incidence of neonatal abnormality (3). Many factors are associated with stress, including the mediators involved in a particular stress response, period of life during which stressful events are experienced (4, 5), intensity of stress and experimental model of either epilepsy or stress (6, 7). Taken together, these findings have indicated that developing brain is susceptible to changes in response to environmental stimuli. Neuropsychological studies have demonstrated that all sensory and motor stimuli entering the central nervous system can cause neuroplasticity changes in brain neurons (8, 9). Moreover, neuroplasticity findings have shown that such stimuli cause an increase in densities of dendritic spines and synaptic connections in brain (10). Another study has indicated that exposure of rodents to music modulates brain development and neuroplasticity (11). It has also been reported that music alters cerebral hemodynamics in human which is a left hemispheric activation in musicians and a right one in non-musicians (12). Initial studies have represented that human fetus reacts to musical stimulation (13, 14), which is called fetal response to music. Some pieces of evidence have suggested that musical training induces significant improvements in abilities such as verbal memory and general intelligence, as demonstrated in the children randomly assigned to either music training, drama or no-training controls (15). It has been also reported that full-term infants’ performance in detection of melodic alterations appears to be influenced by perceptual experience from 6 months to 1 year old. Furthermore, an experiment with prematurely- born infants has supported the hypothesis that music has a positive impact on infant(16). It has been reported that listening to music has a remarkable effect on learning capacity and memory consolidation (17). While many reports have indicated that rat fetuses probably cannot hear by 20^th^ day of gestation (18, 19), it is likely that continuous receiving of musical stimulation would lead to neural plasticity changes in the fetus brain, at least indirectly. It has been reported that classic music induces a fall in plasma prolactin and corticosterone (COS) levels in healthy rats and prevents haloperidol-induced increase. Therefore, it was tested again if exposure to music during gestation reduces corticosterone blood level in current study? Moreover, exposure to music is associated with a significant increase in dopamine levels in many brain areas, especially in prefrontal cortex and substantianigra (20). Moreover, according to the reports, classic music triggers a reduction in systolic pressure and an increase in mesencephalon dopamine levels in the humans and rats treated with ecstasy via a calmodulindependent system (21). Based on these studies from the related literature, it was hypothesized that classic music may affect rat fetuses (at least) through altering maternal neurohumoral factors, such as a reduction in COS and prolactin blood levels. Developing rats exposed to prenatal music showed increased hippocampal neuroplasticity as well as facilitated memory (22). Another study has also showed that music improves visual awareness in neuropsychological patients with visual neglect. This enhancement is possible through the factors associated with the increased activation and functional coupling of the frontal, parietal, and occipital cortical areas involved in emotion, attention, and early vision processing (11). Based on previous suggestions that music shows a significant influence on brain development, we chose to assess neuroplasticity and neuronal density in parietal cortex of fetal rat brain that was prenatally exposed to music. In current study, only parietal cortex was chosen to be investigated because it is the most visible and accessible region of the brain cortex.

## Materials and Methods

In this experimental study, twelve female Wistar rats weighing 180-220 g (10 weeks old on delivery) were obtained from the Animal Facility at Urmia University of Medical Sciences, Urmia, Iran. These rats were housed in groups of three per cage and maintained under the following standard conditions: 12 hour light/dark cycle, 22 ± 2˚C, and food and water *ad libitum*. All the experimental protocols and procedures complied with guidelines of the Declaration of Helsinki (1975), as reflected in the guidelines of Medical Ethics Committee, Ministry of Health, I.R.Iran. In addition, Regional Medical Ethics Committee of West Azerbaijan Province, Islamic Republic of Iran, approved this study. All the females were mated at 12 weeks with a sexually experienced male of the same genotype. Each female was paired with one male at 09:00 and plugged females were checked at 15:00; these females were immediately housed in groups of three per cages for the entire gestation. If a plug was not observed, the animal was returned to her home cage until the next morning for a new mating session. The pregnant rats were divided into two control and musical groups (n=6/ in each group). Musical group was exposed to classic music with 60 dB power for 90 minute twice per day from 2^nd^ to 20^th^ day of gestation (23). Intensity of the background noise in the rearing environment was 32 dB at the time of applying musical stimulus. The control rats were handled similar to those in musical group, but were not exposed to music. Before parturition on the 21^st^ day of gestation, all the dams were deeply anesthetized by ether and two ml of blood samples was collected in ethylene diaminetetracetate (EDTA)-coated tubes. Then, the dams were transcardially perfused by electron microscope (EM) fixative agent and the fetuses were removed, counted, weighed (all the fetuses) and prepared (one fetus per dam, randomly) by 20% glutaraldehyde and 2.5% formaldehyde (Merck, Germany) in the buffered phosphate solution (BPS) (pH=7.5, 0.1 M). The fetal brains (n=6 in each group) were carefully extracted intact and washed three times with the buffered solution. Then, they were dried and fixed using the second tissue fixative, osmium tetroxide, and different degrees of alcohol solutions (50, 70 and 90 %; each for 25 minutes). Afterward, all the specimens were embedded in resin (Epon 812) (Sigma-Aldrich, Missouri, USA) at 60˚C for 24 hours. Parietal cortex was subjected to slice preparation and at least three horizontal slices (semitin and thin sodium) were made using a LKB III ultramicrotome (LKB, Bromma, Sweden) and re-dried with acetate uranyl and lead nitrate for EM (EM LOC, Electron Microscopy Sciences, USA) preparation. The images were obtained from Kodak 4488 films, all of which were observed and analyzed for any possible morphological alterations by an experienced histologist who was blind to the group of the specimens. The blood samples were kept on ice and centrifuged later for 15 minutes at 9000 rpm at 3˚C. Their plasma was transferred to clean 1.5 ml micro-centrifuge tubes and stored frozen at -80˚C until COS levels were determined. This hormone was measured using a commercial enzymelinked immunosorbent assay (ELISA) kit (Cayman, Ellsworth, Michigan, USA).

### Statistical analysis

The data of this experimental study was analyzed using the statistical package for the social sciences (SPSS) (SPSS Inc., Chicago, IL, USA) version 16. The data presenting litter size, body weight, COS level and cell density were normally distributed; therefore, independent t test was applied for their analysis. The results were expressed as mean ± standard error of mean (SEM). Significance level of p<0.05 was considered for all the tests.

## Results

**Fig 1:**
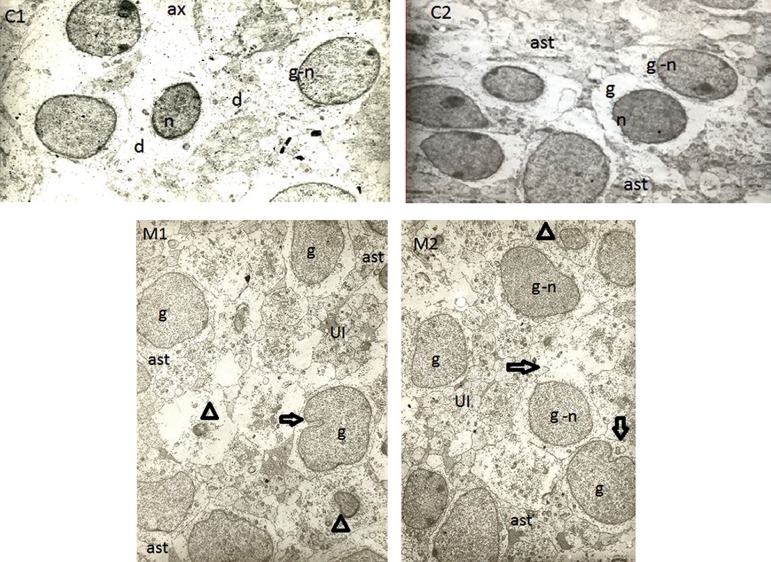
Representative electron microscope (EM) photomicrographs of fetal parietal cortex in rats. The images C1 and C2 belong to two fetuses from control rats, and the images M1 and M2 belong to a music-treated group. Shape of the cells was simpler and smoother in control rats, whereas the cell and nuclear membranes are more complex in the music-treated group. " g" indicates granular cell, " n" stands for nucleus, " ast" indicates astrocyte, " UI" stands for unidentified cells,⇨ indicates indentation in cell membrane and/or nucleus membrane, △ indicates nucleus of unknown cells, " d" stands for dendrite, and " ax" indicates axon.

Microscopic observation indicated that there were remarkable differences between control and musictreated rats. There was morphological difference between the control and musical groups, so that shape of the cells was simpler and smoother in control rats, whereas cell membrane and cytoplasmic organelles were more complex in music-treated group. Moreover, indentation phenomenon in the neuronal cell membrane and nuclear membrane in musical group was higher than the related value in the control. Based on comparing solid structures outside the cells, content of intercellular space was more affluent among music-treated rats than control (Fig 1).

**Fig 2:**
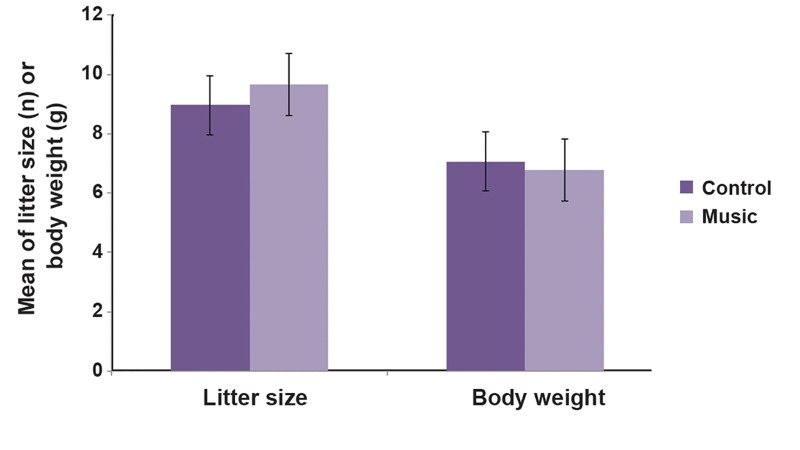
Effect of musical stimuli during fetal period on litter size and body weight in rats. Control rats were maintained intact, but musical group was exposed to music from 2^nd^ to 20^th^ day of gestation. There are no significant differences between the groups in terms of litter size and body weight.

To evaluate effect of prenatal music on density of cortical cells, the cells in an EM field (×50000) were counted based on cells nuclei by a histologist who was blind to the rat groups. This evaluation revealed that there were more cells per EM field (cell density) in parietal cortex of music-treated rats than in control (Table 1). Meanwhile, there was no significant difference in litter size and body weight between the two groups (Fig 2).

To evaluate effect of classic music during gestation on COS blood levels in dams, values of COS were compared between music-treated and control rats. Music significantly reduced COS blood levels in pregnant rats (Table 1).

**Table 1:** Exposure effect to classic music (60 dB) on corticosterone (COS) blood level in dams and cortical cells density in fetus brain of Wistar rat


Groups	Corticosterone (ng/m)	Cell density (cell/EM field)

**Control (n=6)**	37.01 ± 2.58	5.5 ± 0.43
**Music (n=6)**	29.53 ± 1.43**	7.17 ± 0.6 *


Values are means ± SEM. **; P=0.02 and *; P<0.05.

## Discussion

Result of current study indicated that there was significant morphological difference in the cellular and intercellular spaces between the two groups. Also, the music-treated rats had significantly higher cell density in the parietal cortex. Several lines of studies have suggested that neuroplasticity is even present in adult brains. Skills and practice required for a profession appear to induce lasting changes within neural structures, for example professional typists undergo greater development of neural regions related to programming motor tasks, or musicians appear to acquire increased cortical representation of their digits as well as enlarged motor, auditory and visual-spatial regions. Even among the elderly, neuroplasticity continues to facilitate changes, which leads to improvement in cognitive function. These studies have indicated that the largest changes occur through repeated practice of a skill over an extended period of time, even learning in adulthood (15). It has been reported that listening to music has a remarkable effect on learning capacity and memory consolidation (17). Evidence has demonstrated that regular musical leisure activities can have long-term cognitive, emotional and social benefits in mild/moderate dementia and could be, therefore, utilized in dementia care and rehabilitation (24). Another report has indicated that exposure of rodents to music modulates brain development and neuroplasticity by mechanisms that involve facilitated hippocampal neurogenesis, neurotrophin synthesis and glutamatergic signaling (11). An increasing body of reports has started to document impacts of musical stimuli on brain development and neuroplasticity in animal models. To confirm this point, exposure of chicken embryos to music induces increased volumes and neuron densities in brainstem auditory nuclei (25). Other investigations have indicated that perinatal exposure to music efficiently protects spontaneous alternation performance against the deficits induced by callosotomy. These findings may offer significant understandings into music-induced neuroplasticity, applicable to brain development and neurorehabilitation (11). Experience has shown that using music for therapeutic purposes has definite effects on neuropsychiatric disorders and music therapy is presently being administered mostly in western countries in clinical and welfare settings. However, the mechanisms of action underlying music therapy have still remained unknown and no scientific progress has been made (22). Impacts of musical stimuli on neurogenesis might be arbitrated to neurotrophin synthesis in brain. Perinatal exposure to music reduces level of nerve growth factor (NGF) and increases level of brain-derived neurotrophic factor (BDNF) in the hippocampus and hypothalamus of mice. Music stimulation is also associated with higher performance of mice in passive avoidance tasks (11). Findings have also represented that perinatal music stimuli has an effect on glutamate signaling by increasing levels of N-methyl-D-aspartate (NMDA) receptor N-methyl receptor 2B (NR2B) subunit in the auditory cortex as well as á-Amino-3-hydroxy-5- methyl-4-isoxazolepropionic acid (AMPA) receptor glutamate receptor 2 (GluR2) subunit in the auditory cortex and cingulate gyrus of rats (26, 27). Previous study of the present authors showed that prenatal stress induced a significant rise in COS blood level and NMDA receptors density in brain of the offspring (28). The result of the current study was in line with the above-mentioned works, indicating that exposure to music during fetal period could increase cell density and enrich intracellular and interstitial spaces in fetal rat brain cortex. However, litter size and body weight of the fetuses did not change by the music stimuli. Previous studies have indicated that prenatal stress leads to increased COS blood level in dams and pups, LBW and increased susceptibility to pentylentetrazole (PTZ)- and pilocarpine- induced seizure (6, 7, 29). It is likely that impact of music is, at least partly, different from that of prenatal stress; animals might like music stimuli (but not stress) and/or listening to music is not a stressful event for pregnant rats. In the present study, exposure to music seems to calm the dams because COS level was significantly lower in music-treated rats than control (Table 1). It has been reported that rat fetuses probably cannot hear by 20^th^ day of gestational (19, 20); therefore, effect of prenatal music in rats could result in maternal changes, such as decreased COS and prolactin levels (20). Exposure to high level of glucocorticoids in the prenatal period mimics exposure to prenatal stress in rats, which leads to neural injury (30), LBW and increased susceptibility to PTZ- and pilocarpineinduced seizure (6, 7, 29). It seems that decreased COS level could be partly the reason for higher cell density and rapid morphological changes in parietal cortex of music-treated fetuses. Previous studies have been reported that both glucocorticoid receptors (GR) and mineralocorticoid receptors (MR) that are present at fetal rat brain play important roles in physiological system (31-33). Endogenous glucocorticoids and synthetic glucocorticoids exposure have a number of critical effects in the fetal brain, including modification of neurotransmitter systems and transcriptional machinery via these specific receptors (34). Neuropsychological and neurological studies using advanced investigation and diagnosis techniques, such as transcranial Doppler (TCD) sonography, positron emission tomography (PET) scan and functional magnetic resonance imaging (fMRI), have indicated that perception of music is not solely related to right hemisphere, whereas neural network of music perception is widely distributed in both hemispheres. In other words, no music perception center has been identified in the brain, and the whole brain is involved in music (35). Findings have suggested that exposure to music during pregnancy has no effect on body weight, while many studies have indicated that prenatal exposure to noise and/or stress leads to LBW and growth retardation (3, 6, 7, 29). Result of the current study was in line with such investigations, showing no significant difference in terms of body weight between musictreated and control rats. Also, the results indicated no significant difference in terms of litter size between the two groups. To the best knowledge of the present authors, there were no available data regarding effect of music on litter size. Exposure of the rats to music in the current study occurred after mating and formation of zygotes; therefore, music could not affect the number of fetuses. If music is going to have an impact on litter size, it must be applied prior to mating. There are few studies relating to effect of prenatal exposure to music on neurogenesis and brain development, almost all of which have emphasized positive effects of music on neurogenesis and brain development (3, 11, 26, 27). Unique property of the present study was that it was totally conducted during fetal period. As mentioned in the methods section, all the dams were transcardially perfused using EM fixative agent before parturition and the fetuses were then removed and prepared for EM observation. In this study, there was no direct environmental stimulation (extra-uterine stimuli), which might affect the pups because all the fetuses were fixed and extracted before parturition. Therefore, it can be stated that the results were almost completely related to intra-uterine musical effect, which was applied to the dams. Although it was stated that rat fetuses could not hear until the birth time, direct effect of music on fetuses could not be ruled out. It can be recommended that such an effect might be the result of rhythmic vibration of amniotic fluids and/or umbilical cords following musical stimuli, but currently, there is no evidence for this opinion in the case of rats. Confirming this opinion, it has been reported that external sounds to human fetus which are not directly transmitted to the heart or umbilical cord are experienced through generalized vibrations of the amniotic fluid. They are felt all around the body at every extremity. Rhythms associated with predictable and unpredictable movements of mothers or events in their environment are experienced on a regular to irregular continuum (pulse to complex rhythm) (36). Meanwhile, we did not study other parts of brain cortex due to limited funding provided for this study.

## Conclusion

It can be concluded that exposure to long-lasting musical stimulation with favorable intensity and rhythm in fetal period (not stressful for mothers) would have a great impact on general neuroplasticity of fetal rat brain, at least indirectly. Music-induced neuroplasticity may lead to some degrees of improvement in higher functions of brain and alter training ability of the offspring. The findings of this study may open a new horizon regarding the implication of music therapy during gestation for healthy and nonhealthy mothers. Although this study was simple and did not lead to extensive results, it was a pioneer in this context and could shed light on this field of investigation. It can be concluded that effect of music on rat fetus is a mixed phenomenon which is experienced both directly by fetuses and indirectly by the dam.
